# Gitelman’s syndrome complicated by mild renal insufficiency and high anion gap acidosis; a rare presentation in a young female

**DOI:** 10.12860/jnp.2015.08

**Published:** 2015-04-01

**Authors:** Nazrul Hassan Jafry, Ejaz Ahmed, Muhammed Mubarak

**Affiliations:** ^1^Department of Nephrology, Sindh Institute of Urology and Transplantation, Karachi, Pakistan; ^2^Department of Histopathology, Sindh Institute of Urology and Transplantation, Karachi, Pakistan

**Keywords:** Acidosis, Azotemia, Autosomal recessive, Gitelman’s syndrome, Hypokalemia

## Abstract

*Background:* Gitelman’s syndrome (GS) is a rare autosomal recessive renal tubular disorder that is characterized by episodic clinical manifestations and persistent biochemical abnormalities. The disorder manifests in adolescent or adult age and is characterized by transient episodes of muscle weakness and tetany. Its diagnosis requires a high index of suspicion and skillful interpretation of laboratory investigations.

*Case Presentation:* We herein present a case of a 20-year-old female patient who presented with generalized muscle weakness and mild renal insufficiency. Laboratory investigations revealed mild azotemia, high anion gap acidosis, hypokalemia, hypomagnesemia, and hypocalciuria. She recovered her renal functions and muscle power with appropriate management and is doing well seven months after her first presentation to our hospital.

*Conclusions:* This case highlights the need to create high index of suspicion among the general practitioners about this syndrome and an early referral of such patients to nephrologists for an accurate diagnosis and appropriate management.

**Implication for health policy/practice/research/medical education:**

Gitelman’s syndrome (GS) is a rare primary hereditary renal tubular disorder that is characterized by episodic clinical manifestations and persistent biochemical abnormalities. It is also known as familial hypokalemia-hypomagnesemia. It is transmitted as an autosomal recessive trait. Mutations in the solute carrier family12, member 3 gene, *SLC12A3*, encoding the thiazide-sensitive NaCl cotransporter (NCC), are found in the majority of GS patients. In a small number of cases, mutations in another gene, *CLCNKB*, encoding the chloride channel ClC-Kb have been implicated. The disorder manifests in adolescent or adult age and is characterized by transient episodes of muscle weakness and tetany. Its diagnosis requires a high index of suspicion. The long-term prognosis is excellent.

## 1. Introduction

Gitelman’s syndrome (GS) is a rare primary hereditary renal tubular disorder that is characterized by episodic clinical manifestations and persistent biochemical abnormalities (1-5). It is also known as familial hypokalemia-hypomagnesemia. It is transmitted as an autosomal recessive trait. Mutations in the solute carrier family12, member 3 gene, *SLC12A3*, encoding the thiazide-sensitive NaCl cotransporter (NCC), are found in the majority of GS patients. In a small number of cases, mutations in another gene, *CLCNKB*, encoding the chloride channel ClC-Kb have been implicated (6-8). The disorder manifests in adolescent or adult age and is characterized by transient episodes of muscle weakness and tetany (6-8). Its diagnosis requires a high index of suspicion. The long-term prognosis is excellent (9,10).

We herein present a case of a young female patient who presented with generalized muscle weakness and mild renal insufficiency. She had mild azotemia, high anion gap acidosis, hypokalemia, hypomagnesemia, and hypocalciuria. She recovered her renal functions and muscle power with appropriate management and is doing well seven months after first presentation to us.

## 2. Case Presentation

A 20-year-old female, unmarried, was admitted via the emergency department of our hospital on 11th December 2013 with complaints of generalized weakness since 2 weeks, difficulty in swallowing since 2 weeks, and loose stools since 5 days. The generalized weakness was gradual in onset and more marked in legs, so much so that she was unable to rise from the bed. She also felt difficulty in swallowing and in subsequent days, difficulty even voiding urine. She also complained of loose stools 4 to 5 times a day, semisolid in consistency. No mucous and no blood was noticed. However, these were associated with occasional abdominal cramps. In systemic inquiry, she did not complain of fever, sore throat, rashes, joint pain, swelling, cough, chest pain, dysuria, hematemesis or backache.

In the past history, she experienced similar episodes intermittently over the last five years. During these episodes, she used to develop generalized weakness, more of lower limbs, vomiting, and sometimes muscle cramps. With these complaints, she often visited doctors and also required admission in private hospitals, where she was given intravenous (IV) fluids with potassium and her weakness improved. Then she was discharged on potassium containing tablets which would relieve her symptoms. After recovery from the episodes, she used to discontinue oral potassium supplements. She used to remain well for 6 to 8 months before she developed another similar episode and treated similarly. In this way, she recalled 4 to 5 admissions. Record of some investigations seen showed that the predominant abnormality was low potassium level in the serum with normal renal functions.

Drug history was remarkable for tablet NeoK, capsule Esomeprazole, multivitamin syrup and tablet motilium. She is the only daughter with two brothers who enjoy good health. No family history of similar disease was elicited. Parents were non-consanguineous. She studied till ninth class and then left study due to the recurrent illness. She was not addicted to narcotics. She had adequate appetite and sleep.

On examination, she had short stature, lean built, and looked wasted and sick. Her pulse was 88 beats per minute, blood pressure (BP), 90/60 mm Hg, temperature, 98.6°F and respiratory rate, 20 breaths per minute. She was not pale looking, icteric or cyanosed. No koilonychia or clubbing was noted. Dehydration was positive, and muscles were wasted.

Her abdomen was soft, non tender, with palpable bladder but no other visceromegaly. The chest was clear. Both heart sounds were audible in all four areas. No added sounds were noted. In central nervous system (CNS) examination, Glasgow coma scale (GCS) was15/15; power was 3/5 in both lower limbs, 4/5 in upper limbs with intact sensation. Deep tendon reflexes were diminished in all 4 limbs.

A clinical differential of malabsorption, periodic hypokalemic paralysis, or salt-losing nephropathy was contemplated.

On laboratory investigations, serum urea was 73 mg/dl; creatinine, 1.72 mg/dl; sodium, 127 mEq/L; potassium, 1.4 mEq/L; chloride, 101 mEq/L; and bicarbonate, 09 mEq/L. Her hemogram showed hemoglobin (Hb) of 15.1 g/dl; total leucocyte count (TLC), 37.4×10^9^/L; and platelets, 494×10^9^/L. Urine analysis revealed pH of 7.00; albumin, 2+; pus cells, occasional; and red blood cells (RBCs), numerous. Corrected anion gap was 22. Further biochemical tests showed serum calcium, 6.4 mg/dl; phosphorus, 3.2 mg/dl; serum albumin, 1.8 g/dl; total bilirubin, 0.86 mg/dl; direct bilirubin, 0.08 mg/dl; alkaline phosphatase, 50 IU/L; serum glutamic oxaloacetic transaminase (SGOT), 14 IU/L; serum glutamic-pyruvic transaminase (SGPT), 6 IU/L; and gamma-glutamyl transferase (GGT), 7 IU/L.

**Figure 1:**
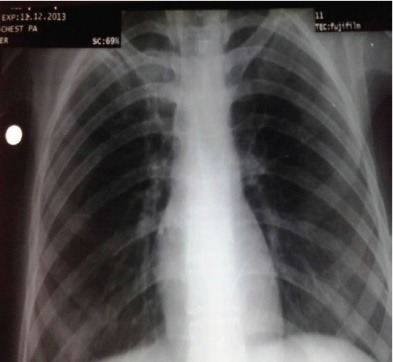
X-ray chest showing no significant abnormality in lung fields or heart.

**Figure 2:**
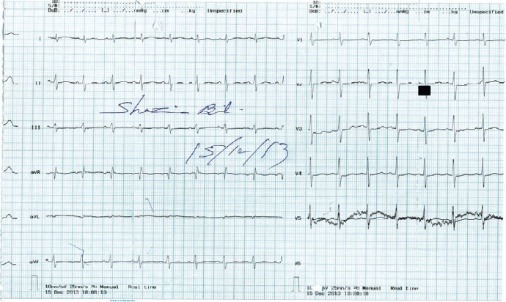
Electocardiograph showing typical changes of hypokalemia.

Ultrasound abdomen showed both sided normal size kidneys with mild pelvicalyceal dilatation bilaterally. Bladder was distended with approximate volume of 430 ml. Chest X-ray was done, which was unremarkable (Figure 1). Electrocardiography done showed flattening of T waves with occasional inversion in some leads, findings typically seen in hypokalemia (Figure 2).

She was catheterized, rehydrated with isotonic saline with KCL, and IV antibiotics started on suspicion of urinary tract infection (UTI) as TLC was high. She maintained good urinary output (>1500 ml). By admission day 5, diarrhea had settled. Repeat laboratory tests at this time showed: serum urea of 47 mg/dl; creatinine, 1.6 mg/dl; potassium, 2.5 mEq/L and sodium, 127 mEq/L.

**Figure 3:**
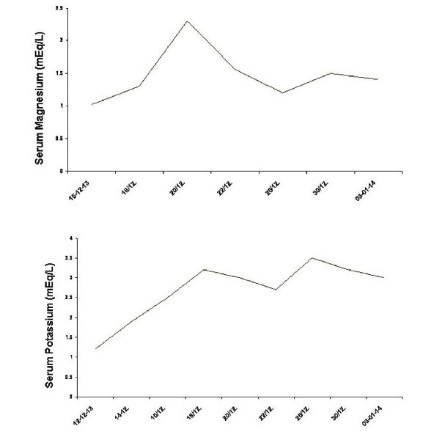
Line graph showing serial levels of serum magnesium and potassium during admission period.

Further investigations showed serum magnesium of 1.02 mg/dl. IV magnesium sulphate was administered daily with modest elevation in serum levels during subsequent days. Potassium requirement remained high and was administered by oral and IV routes. On admission day 10, her serum creatinine was 0.85 mg/dl; potassium, 2.6 mEq/L; and magnesium, 0.95 mg/dl. The temporal changes in serum magnesium and potassium are shown in Figure 3. The 24-h urinary metabolic study on day 10th of admission showed potassium, 44 mmol/24h; magnesium, 50 mg/ 24 h; calcium, 108 mg/24 h (normal range: 100 to 300 mg/24 h). Fractional excretion of magnesium was 27.3%. In the face of low serum magnesium, fractional magnesium excretion should be less than 2%.

Her gastrointestinal (GI) workup was also done including upper GI endoscopy and sigmoidoscopy. Upper GI endoscopy revealed normal mucosa, with mild antral erythema. Sigmoidoscopy showed normal mucosa. Tissue biopsies were also obtained. The duodenal biopsy showed mild stunting of villi with slightly increased intraepithelial lymphocytes; the antrum showed mild chronic nonspecific gastritis and the rectosigmoid, mild nonspecific colitis.

Anti-trangultaminase antibodies and anti-deamidated gliadin-derived peptide antibodies were negative. Final diagnosis was made of GS along with changes of tropical sprue on duodenal biopsy. Genetic testing could not be done due to non-availability of the test.

She was discharged on third January 2014 on tablet spironolactone 25 mg, once daily, increased subsequently to 50 mg; tablet Neok, 2 tablets, 8-hourly, capsule magnesium chloride 1 capsule, 8-hourly, tablet folic acid 5 mg, once daily, tablet Ciproxin 250 mg, 12-hourly. Laboratory tests on 2-week follow up showed serum urea of 15 mg/ dl; creatinine, 0.4 mg/dl; sodium, 142 mEq/L; potassium, 3.6 mEq/L; chloride, 102 mEq/L; bicarbonate, 30 mEq/L; calcium, 8.8 mg/dl; phosphorus, 3.8 mg/dl; albumin, 3.1 g/dl; and magnesium, 1.9 mg/dl. She is on regular follow-up and doing well at seven months of follow-up.

## 3. Discussion

GS is a hereditary renal tubulopathy characterized by hypokalemic metabolic alkalosis in association with significant hypomagnesemia and hypocalciuria (1-5).

Our case highlights a number of important points about this syndrome. Although the syndrome typically does not cause renal insufficiency, our patient presented to us with mild renal insufficiency. This was most probably due to prerenal azotemia resulting from diarrhea and reduced oral intake in our patient. This also resulted in high anion gap metabolic acidosis, which is unusual finding in this syndrome. GS typically causes metabolic alkalosis.

The other point which is worth highlighting is the underdiagnosis or misdiagnosis of the condition. Our patient presented to different general practitioners who probably did not make the correct diagnosis and counsel the patient or the family. They treated the patient symptomatically and upon improvement stopped the medications. This resulted in repeated attacks of the syndrome in our patient. During this presentation, she also developed azotemia, which prompted her doctors to refer her to our hospital for diagnosis and management.

Clinically, the disease can be asymptomatic or manifest in a number of ways. In the majority of cases, symptoms do not appear before the age of six years; the disease usually being diagnosed during adolescence or adulthood. Transient episodes of muscle weakness and tetany, sometimes accompanied by abdominal pain, vomiting and fever are common features. Our patient had all the classical presenting features of the syndrome. In addition, during the present attack, she also developed pre-renal azotemia, which has not been reported previously. Some patients present for the first time in adult age with chondrocalcinosis resulting in swelling, local heat, and tenderness over the affected joints. Rare complications include sudden cardiac death and growth retardation in severe cases (1-8).

Diagnosis is based on the combination of clinical symptoms and biochemical abnormalities (hypokalemia, metabolic alkalosis, hypomagnesemia and hypocalciuria). It also requires exclusion of other causes of potassium and other electrolyte losses. We also worked up our patient for GI pathology, which revealed tropical sprue on duodenal biopsy. She showed improvement in her diarrheal illness with the use of antibiotics.

Genetic counseling is important for patients and families of GS patients. Antenatal diagnosis for GS is possible but usually not recommended because of the good prognosis in the majority of patients (8-10). We also counseled the patient and the family regarding lifelong nature of the problem and regular follow-up and use of medications. However, genetic testing was not done due to non-availability of the test.

Management is primarily aimed at correcting electrolyte deficiencies with some promising effects of indomethacin and anti-aldosterone drugs. Most asymptomatic patients with GS remain untreated and undergo ambulatory monitoring, once a year, usually by nephrologists. Lifelong supplementation of magnesium and potassium is recommended along with indomethacin or spironolactone or amiloride. In general, the long-term prognosis of GS is excellent.

## 4. Conclusions

This case highlights the need to create awareness among the general practitioners about this syndrome and referral of such patients to nephrologists for an accurate diagnosis and appropriate management.

## Authors’ contributions

All authors contributed to the manuscript equally.

## Conflict of interests

The authors declared no competing interests.

## Funding/Support

None.
